# Snapshot 3D Electron Imaging of Structural Dynamics

**DOI:** 10.1038/s41598-017-10654-x

**Published:** 2017-09-07

**Authors:** Liu-Gu Chen, Jamie Warner, Angus I. Kirkland, Fu-Rong Chen, Dirk Van Dyck

**Affiliations:** 1 National Tsing-Hua University, Department of Engineering and System Science, Hsin-Chu, Taiwan; 2 University of Oxford, Department of Materials, Oxford, OX1 3PH UK; 3 Electron Physical Sciences Imaging Centre, Diamond Light Source Ltd, Harwell Science & Innovation Campus, Didcot, Oxfordshire OX11 0DE UK; 4 University of Antwerp, EMAT, Department of Physics, B2020 Antwerp, Belgium

## Abstract

In order to understand the physical properties of materials it is necessary to determine the 3D positions of all atoms. There has been significant progress towards this goal using electron tomography. However, this method requires a relatively high electron dose and often extended acquisition times which precludes the study of structural dynamics such as defect formation and evolution. In this work we describe a method that enables the determination of 3D atomic positions with high precision from single high resolution electron microscopic images of graphene that show dynamic processes. We have applied this to the study of electron beam induced defect coalescence and to long range rippling in graphene. The latter strongly influences the mechanical and electronic properties of this material that are important for possible future applications.

## Introduction

For studies of defect dynamics in graphene and related 2D materials there is a need for a method that can recover time resolved three-dimensional information in real space. Electron tomography has been demonstrated using aberration corrected Scanning Transmission Electron Microscopy (STEM)^1, 2^ and Transmission Electron Microscopy (TEM)^3, 4^, but this method requires a large number of images making it unsuitable for the observation of dynamic processes.

In this letter we present a method that can be used to determine the 3D positions of individual atoms in a graphene sheet from a single TEM image and subsequently to track their motion. This “snapshot’ method provides the four dimensional information (three spatial and the time domain (*x, y, z, t*)) necessary for the understanding of certain dynamic processes. As an example, the interaction of high-energy electrons with monolayer graphene causes variations in both bond lengths and angles leading to elastic deformations and the migration of defects^5, 6^. We report initial applications of this approach to studies of the long range rippling of monolayer graphene and to defect coalescence and motion.

The discovery of graphene as the first truly 2D crystal contradicted the Mermin–Wagner theorem^7^ which states that 3D fluctuations can destroy long-range order in 2D crystals. Subsequent theoretical calculations have shown that a monolayer graphene sheet is stabilized by 3D rippling with amplitudes of order less than 0.1 nm and wavelengths of order 8 nm^8, 9^. It has also been reported that the rippling amplitude depends on the size of individual graphene flakes^10^. Experiments, using electron diffraction of μm sized flakes of monolayer graphene have revealed rippling attributed to thermal fluctuations with a wavelength of order 5–10 nm but with amplitudes estimated of order 1nm^10^. Additional studies using TEM^11^ and STEM^12, 13^ imaging have also revealed local rippling in mono and multi layer graphene and distortions around defects^14^ and tears and cracks in graphene sheets^15^. Understanding rippling dynamics is crucial for detailed descriptions of stability and electronic transport^16, 17^ in graphene. Dislocation induced rippling has also been measured using a geometric phase analysis^18^.

## Results

### Experimental conditions for imaging

**Figure 1:**
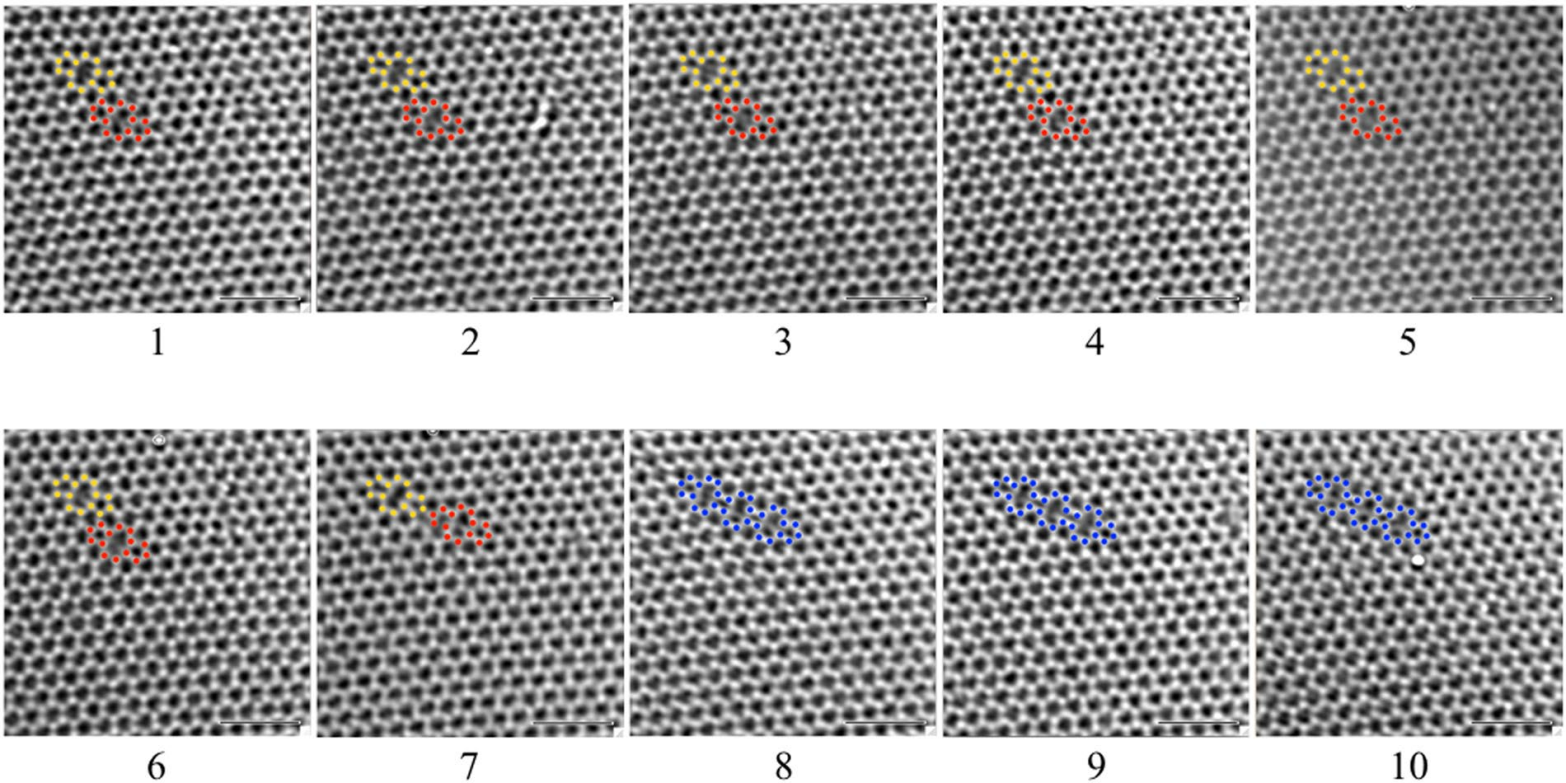
Time series of HRTEM images of graphene. Ten image sequence recorded at an accelerating voltage = 80 kV, with 1 s intervals and with a dose = 10^4^e^−^nm^−2^ for each image. Atoms around defect sites are highlighted in colour; red and yellow show respectively the initial 5-8-5 ring defects and blue the merged 5-8-4-8-4-8-5 extended defect. The scale bar is equivalent to 1 nm. Each image field contains approximately 500 carbon atoms.

Graphene was synthesised by atmospheric pressure chemical vapour deposition (CVD). Full details of the synthesis and transfer onto TEM specimen grids are described elsewhere^19^. Our method was applied to a time-series of ten TEM images (Fig. 1 and Extended data Fig. 1) of graphene flakes supported on perforated Si_3_N_4_ membranes, recorded with plane wave illumination using the aberration corrected Oxford-JEOL JEM-2200MCO FEGTEM^20, 21^ on a 4k × 4k CCD at an accelerating voltage of 80 kV, using an energy spread of 2220 meV and with a dose of 10^4^e^−^nm^−2^. The dose used is higher than that reported for imaging MoS_2_ nanosheets^22^ and was limited by the requirement of recording images with sufficiently high signal to noise for further analysis. The sampling was 0.00861 nm per pixel and the exposure used was 1 second. During data acquisition the CCD gain, and dark field correction remained constant. The field of view containing *ca*. 500 carbon atoms in each frame, was *ca*. 3.5 nm × 3.5 nm and frames were recorded at *ca*. 1 s intervals with a 1 s exposure. Under certain conditions HREM images of a planar thin sample contain 3D information about the sample in that the vertical position of an atom affects the intensity in the image, equivalent to a defocus offset.

### Determination of the *z* height

The algorithm used to determine the z-height of single atom is based on the projected charge density (PCD) approximation^23, 24^ which is valid for the material studied. Vertically displacing a carbon atom, considered as a phase object, over a distance *Δz*, in the presence of small values of high order residual aberrations, gives rise to a change in image intensity, Δ*I* which is dependent only on defocus and can be described by the PCD approximation^23, 24^ (1), where the *λ* is the wavelength of incident electron, *σ* is an interaction constant, *ε*_*o*_ is the permittivity of vacuum, *ε* the relative permittivity and *ρ(x, y)* is the projected charge density which is related to the projected potential *V(x, y)* by the Poisson equation.

1 $${\rm{\Delta }}I(x,y,{\rm{\Delta }}z)={\rm{\Delta }}z(\lambda \sigma /2\pi )(\rho (x,y)/\varepsilon {\varepsilon }_{o})$$

In (1), *ΔΙ* is the experimental intensity after subtraction of the background intensity (measured in the center of the hexagonal ring of C atoms). Hence, using (1) the vertical displacement of an atom, *Δz* can be determined using the change in *ΔΙ* and a calculated value of *ρ(x, y)*. A calculated value for *ρ(x, y)* can be evaluated from a plot of simulated values for *ΔΙ* calculated using the multislice method^24–26^ for a graphene monolayer layer at defoci from 0 to 0.5 nm (blue line in Extended data Fig. 2). For these calculations g_max_ = 20 nm^−1^ corresponding to the experimental information limit and from (1) the fitted slope is proportional to *(λσ/2π) ρ(x, y)/εε*_*ο*_, where *ρ(x, y)/εε*_*ο*_ is 70.9 eVÅ^−1^ in this case.

In principle, the analysis described above could be applied to thicker samples such as bilayer graphene, provided that the underlying projected charge density (PCD) approximation remains valid. However, to calculate a general upper thickness bound for the PCD approximation is materials dependent and would require extensive image simulations which are outside the scope of the current paper.

### Experimental Errors

Experimentally, measurements of the image intensity are affected by the presence of noise, residual aberrations and sample tilt and therefore these factors also limit the precision of this approach in the determination of *Δz* (see also Methods-I and II and Extended data Figs 2 and 3). In addition, the mean displacement squares < u^2^ > , and hence the Debye-Waller factors of carbon atoms at defect sites compared to those in the pristine graphene lattice are expected to be different as atoms at defect sites are less coordinated, which may further affect the precision of this approach (for details see also Methods-III and Extended data Fig. 4).

For an aberration corrected instrument the residual higher order aberrations are small and hence their contribution to the measurement error is negligible (~3 pm, see also Methods-I) compared to that due to noise in the data. The intensities in images of single layer graphene are also less sensitive to the effects of sample tilt as the reciprocal space rel-rods are extended compared to those in a thicker crystal. Sample tilt introduces a globally inclined defocus plane which is automatically accounted for by equation (1). Sample tilt can however also introduce atom displacements and distortions of the local hexagonal symmetry in the case of double layer graphene used to validate our calibration of the Stobbs factor (see also Methods-II). Overall, as will be described later residual aberrations, sample tilt and Debye Waller factors lead to a combined error of 3–7 pm in the determination of the z-height. It is worth also noting that the electron beam interaction may soften the potential^4^ and therefore affect the determination of *Δz*. In this work the relatively high dose used was driven by a need to record data with sufficiently high signal to noise to validate the method described. However, in future using high speed direct electron detectors it should be possible to work under lower dose conditions which will provide useful insights into true structural kinetic effects.

**Figure 2:**
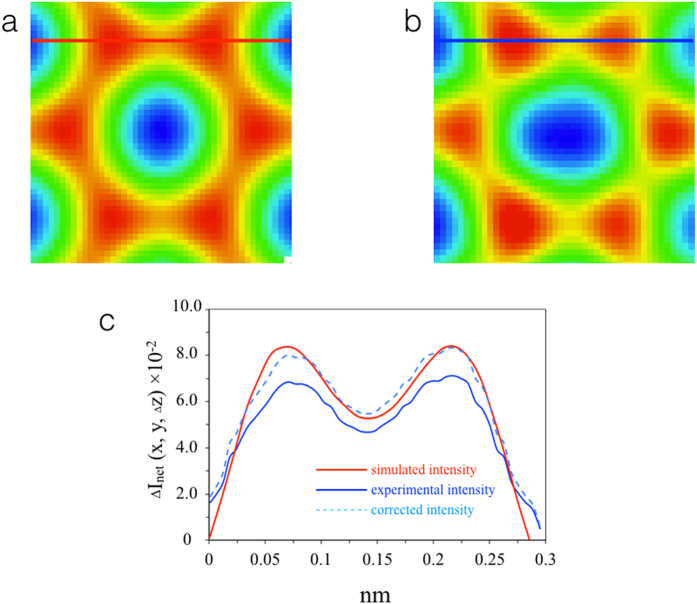
Calibration of the Stobbs factor: **(a)** Simulated and (**b)** Experimental images of an averaged 6 carbon atom motif. (**c)** Intensity profiles along the line marked in (**a**) and (**b**).

It has been established that even in the absence of residual aberrations and specimen tilt there is a contrast mismatch between experimental and calculated high resolution TEM images known as the Stobbs factor^27–29^. Hence, using calculated values of *ρ(x, y)* and experimental values of *ΔI* leads to a systematic error in the calculation of *Δz*. However, for the case of monolayer graphene for which the thickness is known exactly the Stobbs’ factor can be calibrated by quantitatively comparing simulated and experimental image intensities (Fig. 2). Before calibration of the Stobbs factor S = *I*_*sim*_/*I*_*exp*_ the simulated or experimental background intensity, respectively, in the center of the hexagonal ring of C atoms was subtracted from the intensities I_sim_ and I_exp_. Figure 2a and b show the background subtracted simulated image at *Δz* = 0 and the corresponding experimental motif averaged from different locations in the single layer region. The Stobbs factor, *S* was then measured from the intensity ratio between simulated and averaged experimental motifs *I*_*sim*_ */I*_*exp*_ (Fig. 2). The respective intensity profiles are shown in Fig. 2c. This Stobbs factor *S* was then used to correct the simulated PCD to obtain an estimate for the experimental PCD. For the data reported here, *S* = 1.18 and this value was subsequently used to calibrate the vertical displacements.

**Figure 3:**
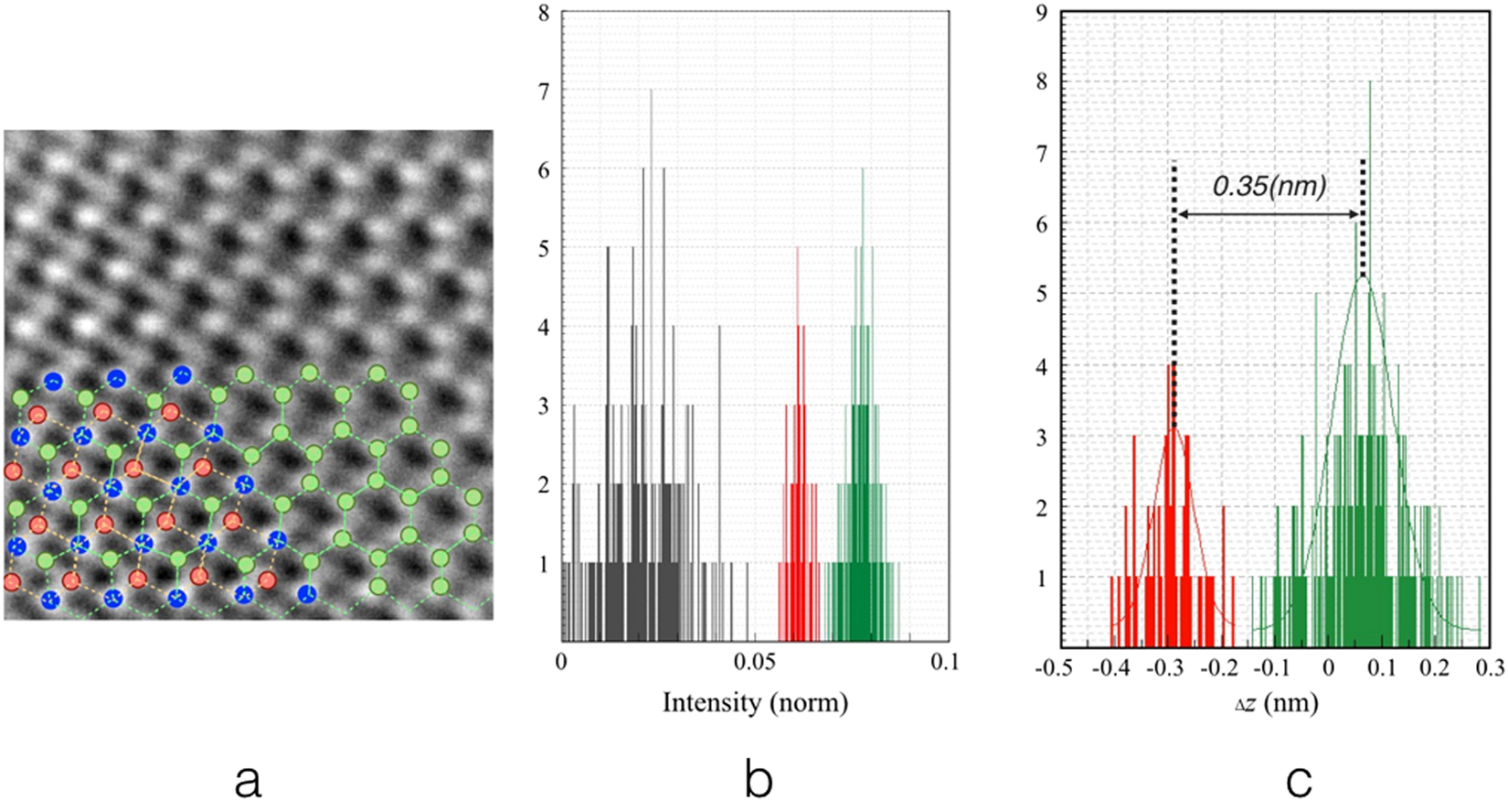
Validation of the calibration. (**a)** Image of graphene showing both monolayer and bilayer regions. Green dots indicate atoms in the bottom layer and blue and red dots indicate atoms in the top layer. Atoms indicated in red are those not superimposed with atoms in the bottom layer. Recorded with a sampling of 0.00861 nm per pixel, exposure time = 1 s, dose = 10^4^ e^−^nm^−2^ at 80 kV. (**b)** Histogram of intensities at the centres of the hexagonal ring of C atoms (valley black, from 3106 pixels), top layer (red, 55 atoms) and bottom layer (green, 316 atoms). (**c**) Histogram of vertical displacements of atoms in the top layer (red) and bottom layer (green).

The validity of this calibration was confirmed experimentally using a sample region containing a flat double-layer graphene sheet (Fig. 3) which has a theoretical spacing between the layers of 0.335 nm and which acts as a control. Within the image field shown there are three types of atom: those in the bottom layer that are not superimposed with those in the upper layer (green dots), those in the upper layer that are not superimposed with those in the bottom layer (red dots) and those in the two layers that are superimposed (blue dots, brightest peaks) (Fig. 3(a)). There is evidence for some atom displacement from perfect hexagonal symmetry in the region shown in Fig. 3(a) suggesting either small local specimen tilt or small residual aberrations. However, as already described (See also Methods I and II) neither of these have a significant effect on the calibration accuracy. In total, 55 and 316 atoms were analyzed, for the top and bottom layers respectively and their intensity histogram and corresponding *Δz* calculated from equation (1) are shown in Fig. 3b and c, respectively.

The error in the measurement of the vertical displacement arising from noise in the data was statistically estimated as follows. Extended data Fig. 5a shows an experimental image of single carbon atom. The position of this atom was located using a fitted 2D Gaussian function and the intensity was extracted from a 3 × 3 pixel area (Extended data Fig. 5b. The quantity *δ(ΔI)* is defined as the deviation in the experimental intensity, *ΔI*_*exp*_ from the fitted Gaussian intensity, *ΔI*_*ideal*_ and hence, the error in *Δz, δ(Δz)* can be calculated using equation (1). Extended data Fig. 5c shows *δ(Δz)* for 20 atoms. From the spread in this data we estimate that the error in the vertical displacement due to noise is 23 pm. In contrast, the errors due to residual aberrations, the Debye Waller factor and sample tilt are estimated to be 3–7 pm (see Methods-I, II and III), which are much smaller than that due to noise. Overall our measured value of the vertical separation in the bilayer region is 0.353 ± 0.03 nm which is in good agreement with the theoretical value of 0.335 nm. The atomic vibrations in graphene are significantly faster than the acquisition time of 1 second. Therefore, the experiments reported measure a “time averaged” value of *Δz* within a 1 s period with an error bar of 30 pm. Even at this time resolution there is evidence for a time dependence in *Δz* for individual atoms. Extended data Fig. 5d shows this time dependent motion in the z-direction for a single atom within a time-series of images in which the measured changes in *Δz* between some members in the series is greater than *δ(Δz)*.

## Discussion

**Figure 4:**
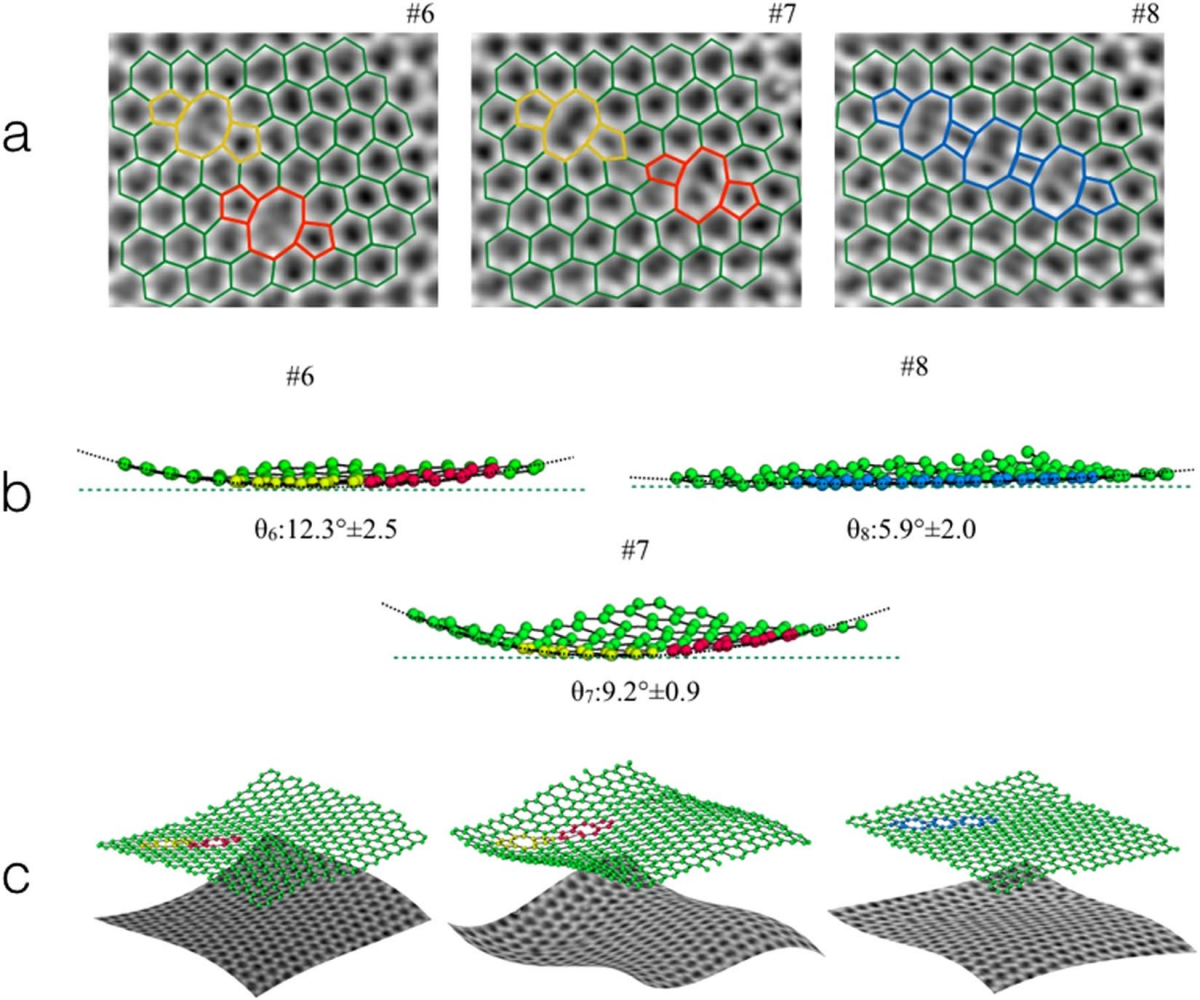
3D analysis of local defect structures. (**a)** Enlarged views of sub-regions from images 6, 7 and 8 in the time series showing the interaction of two defects together with superimposed structure models. (**b)** Rippled structure of the defects in frames 6, 7 and 8 together with the fitted local curvature of the graphene lattice and associated errors. (**c)** Visualization of long range oscillations of the extended graphene sheet using a hyper-surface fitted to the experimental data. The z-height is enlarged 10 times for clarity.

Figure 4(a) shows projected structures and superimposed models of two defects present in sub areas extracted from three images within the ten-member series. These defects are composed of 5 and 8-member rings as previously reported^5, 6, 30^. These two defects are stable in frames 1–5 (Fig. 1 and Extended data Fig. 1), and subsequently migrate in frames 6 and 7 and finally merge in frame 8 to form an extended line defect containing 4-member rings^30^ which is stable in frames 9 and 10 (Fig. 1 and Extended data Fig. 1).

Using the method as described these images of graphene were analysed to determine (*x, y, z*) for each carbon atom around the defect sites shown in Fig. 4(a), as shown in Fig. 4(b) and (c). The z height histograms for frames 6, 7 and 8 are shown in Extended data Fig. 6. A third order polynomial *f(x, y)* was used to fit the three dimension coordinates of the carbon atoms to a hyper-surface with, *f(x, y)* the *z*-height values determined from the experimental data and with the two dimensional atomic positions *(x, y)* determined from fitted Gaussian functions. This shows that the defects are initially non-planar and the local curvature of the graphene lattice around the defect is reduced as the defects merge. The final extended defect is approximately planar in agreement with previously reported Density Functional Theory calculations^30^.

Time dependent variations in the bond lengths and bond angles at each C-C pair have also been measured and compared with those in the perfect graphene lattice for each frame in the time sequence of images (Extended data Fig. 7). This reveals that the bond lengths and angles around the 8-fold rings are distorted compared to the perfect lattice in agreement with previously published calculations^30^, consistent with the defect region accommodating elastic strain.

Using the same image series, we have also measured the long range rippling of the extended graphene sheet. From measurements of the vertical positions of all atoms in the images it is clear that the sheet oscillates (Fig. 4(c)). These oscillations have been analysed as a function of time for the ten-member image series (Extended data Fig. 8), giving values of 0.03 ± 0.005 nm and 6.96 ± 0.66 nm respectively for the amplitude and wavelength in close agreement with previously reported experimental and simulated data from a similar sample size^8, 10–13^. Movies showing the dynamics of these oscillations are provided in Supplementary information (Supplementary information Movie 1).

## Conclusions

In this letter we have described a method for the determination of temporarily resolved 3D atomic positions from a series of single high resolution TEM images. Currently, the temporal resolution was limited by the acquisition and readout times of the conventional CCD detector used^31^, but using currently available direct CMOS sensors^32^ this can be extended to ms time intervals. The use of this type of detector operating in a counting mode will also enable lower electron dose rates whilst maintaining a sufficient signal to noise ratio in the data, potentially enabling studies of intrinsic structural changes, under conditions where beam induced effects have been shown to be suppressed^22^. We have initially applied this to the study of electron beam induced defect coalescence and to long range rippling in graphene, both taking advantage of a defined number of single atom species, in projection in monolayer and bilayer sheets to quantify the vertical displacements. However, the methods described could be applied to other 2D materials provided that the underlying projected charge density approximation remains valid which will ultimately set an upper bound on sample thickness.

## Methods

### Contributions of Residual Aberrations to the Image Intensity

The residual aberrations present in the experimental data were measured during corrector adjustment as; A_1_ = 2.2 nm, A_2_ = 23.25 nm, A_3_ = 278.2 nm, B_2_ = 26.34 nm and C_3_ = −1.162μm giving rise to an overall phase shift *χ(* ***g*** *)* given by:

2 $$\begin{array}{c}\chi ({\bf{g}})=Re\{(2\pi /\lambda )[(1/2)({A}_{1}){\lambda }^{2}{{\boldsymbol{g}}}^{{\ast }^{2}}+(1/2)({C}_{1}){\lambda }^{2}{\boldsymbol{g}}{\boldsymbol{g}}\ast \\ \quad \quad \quad +(1/3)(A2){\lambda }^{3}{{\boldsymbol{g}}}^{{\ast }^{3}}+(1/3)(B2){\lambda }^{3}{{\boldsymbol{g}}}^{{\ast }^{2}}{\boldsymbol{g}}\\ \quad \quad \quad +(1/4)(A3){\lambda }^{4}{{\boldsymbol{g}}}^{{\ast }^{4}}+(1/4)(S3){\lambda }^{4}{{\boldsymbol{g}}}^{{\ast }^{3}}{\boldsymbol{g}}+(1/4)(C3){\lambda }^{4}{{\boldsymbol{g}}}^{{\ast }^{2}}{{\boldsymbol{g}}}^{2}]\}\end{array}$$

The image intensity (Extended data Fig. 2) due to the above residual aberrations can be also be calculated using the multislice method^24–26^ and compared to the intensity (Extended data Fig. 2) in the absence of these aberrations. Extended data Fig. 2 shows that the experimental values for A_1_, A_2_, B_2_ and C_3_ leads to only a small change δ(ΔI) in the slope of *ΔΙ* vs *Δz* (Equation 1) corresponding to an error of 3 pm in the determination of *Δz*. The parameters used for the multislice simulations are given in Extended Data Table I.

### Contribution of Sample Tilt to the Image Intensity

To examine the effects of sample tilt on the image intensity simulated images of bilayer graphene at zero and five degrees tilt were calculated using the multislice method^24–26^ as shown in extended data Fig. 3a and b. Extended data Fig. 3c plots these simulated intensities, in the absence of residual aberrations *vs*. tilt angle for atoms in the top and bottom layers. From the vertical positions, *Δz* of the top and bottom atoms calculated using equation (1) the separation *d*_*cal*_ can be calculated as the difference in height. The error in the layer spacing *δd* = *d*_*cal*_ − *d*_*ideal*_ as a function of tilt angle is given in extended data Fig. 3d and is of order 3 pm.

### The Effect of Debye-Waller Factors on the Image Intensity

The mean displacement, <u^2^> of carbon atoms at the sample edge and in a perfect hexagonal ring have been measured independently from a reconstructed exit wave of a monolayer graphene sheet (Extended data Fig. 4a) using a previously reported method^33^. The value of <u^2^> averaged from typically 6000 atoms is *ca*. 150pm^2^ which is in good agreement with the value (~120pm^2^) measured using electron diffraction^34^. Histograms of <u^2^> from atoms in pristine hexagonal rings (190 atoms) and at the edge (130 atoms) where the atoms are under coordinated are given in extended data Fig. 4b. The value of <u^2^> of atoms at the edge extends to 0.2 Å^2^ as edge atoms are under coordinated. However, <u^2^> values of atoms in the defect sites and in pristine regions are indistinguishable. The values of the <u^2^> from the defect regions can be calculated^35^ for a temperature range 100–300 K, and with a defect concentration of 0.05% to 0.1%, <u^2^> lies between 0.05Å^2^ and 2Å^2^. We have quantitatively evaluated the effect of variations in the Debye-Waller factor on the image intensity δ(ΔI) with a reference, ΔI using <u^2^> = 0.01 Å^2^ as shown in extended data Fig. 4c which gives a maximum error in ΔI, corresponding ton <u^2^> = 2.0 of 7.2 pm in the determination of Δz.

## Electronic supplementary material

Supplementary Information

Supplementary Video
