# Supplementary material for: Snapshot 3D Electron Imaging of Structural Dynamics
Source: Sci Rep. 2017 Sep 7;7:10839. doi: 10.1038/s41598-017-10654-x (PMC5589962; doi:10.1038/s41598-017-10654-x)
Supplement: Supplementary file 1 — Supplementary Information [file 41598_2017_10654_MOESM1_ESM.doc]

**Snapshot 3D Electron Imaging of Structural Dynamics**

Liu-Gu Chen^1^, Jamie Warner^2^, Angus I. Kirkland^2, 3*^, Fu-Rong Chen^1*^, Dirk Van Dyck^4*^

1. National Tsing-Hua University, Department of Engineering and System Science, Hsin-Chu, Taiwan

2. University of Oxford, Department of Materials, Oxford OX1 3PH, UK

3. Electron Physical Sciences Imaging Centre, Diamond Light Source Ltd, Harwell Science & Innovation Campus, Didcot, Oxfordshire, OX11 0DE.

4. University of Antwerp, EMAT, Department of Physics, B2020 Antwerp, Belgium

* To whom correspondence should be addressed

| **Model Size**  **(nm x nm x nm)** | **Slice Thickness (nm)** | **Voltage (kV)** | **C_3_(mm)** | **Convergence Angle (mrad)** | **Focal Spread (nm)** | **Scattering factor** | **Limiting Objective Aperture (nm^-1^)** |
| --- | --- | --- | --- | --- | --- | --- | --- |
| **4.51 x 4.51 x 02** | **0.05** | **80** | **-0.0016** | **0.1** | **0.25** | **Doyle Turner** | **8.7** |

**Extended data Table. 1. Multislice imaging calculation parameters.**

**
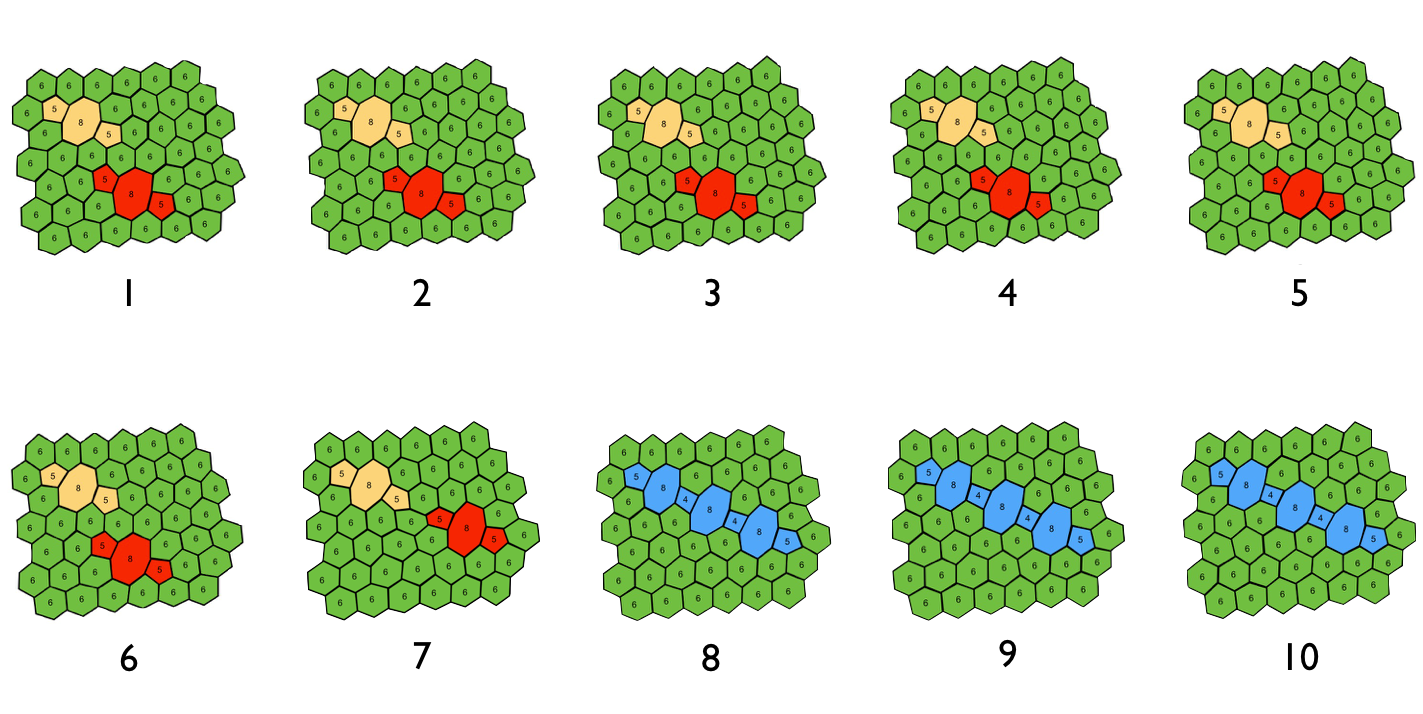
**

**Extended data Fig. 1.** Models of the atomic arrangement around two defects corresponding to the images in Fig. 1. The red and yellow defects contain 5-and 8-member rings (5-8-5 defects). Two defects are stable in 1-5 then migrate towards each other in 6 and 7 and finally merge in 8 to form an extended 5-8-4-8-4-8-5 line defect (blue) containing 4-member rings^22^ which is stable in 9 and 10.

**
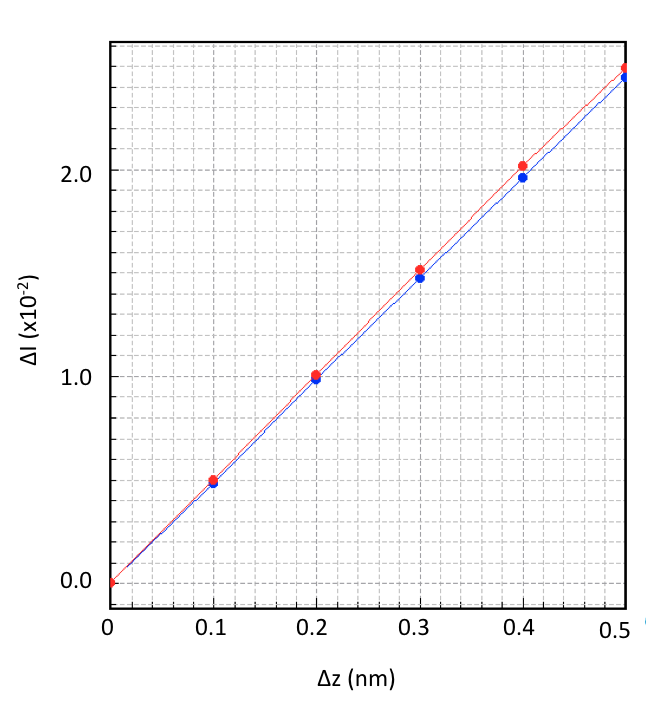
**

**Extended data Fig. 2.** Simulated intensity of a carbon atom in a monolayer graphene sheet as a function of defocus value, *Δz* calculated using the multislice method. The intensity is extracted at the central pixel of the atom. The blue and red lines show simulated intensities without and in the presence of residual aberrations, respectively. The residual aberrations as measured experimentally are A_1_=2.2 nm, A_2_=23.25 nm, A_3_=278.2 nm, B_2_=26.34 nm and C_3_=-1.162 μm. The contribution to ΔΙ from these residual aberrations gives rise to an error in Δz of 3pm (Equation 1).


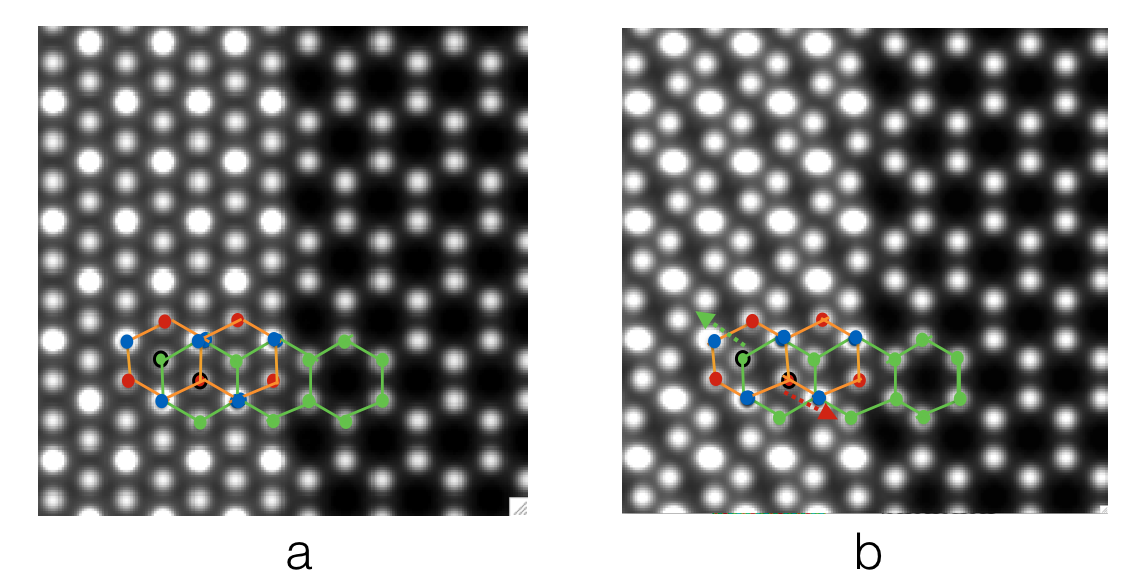


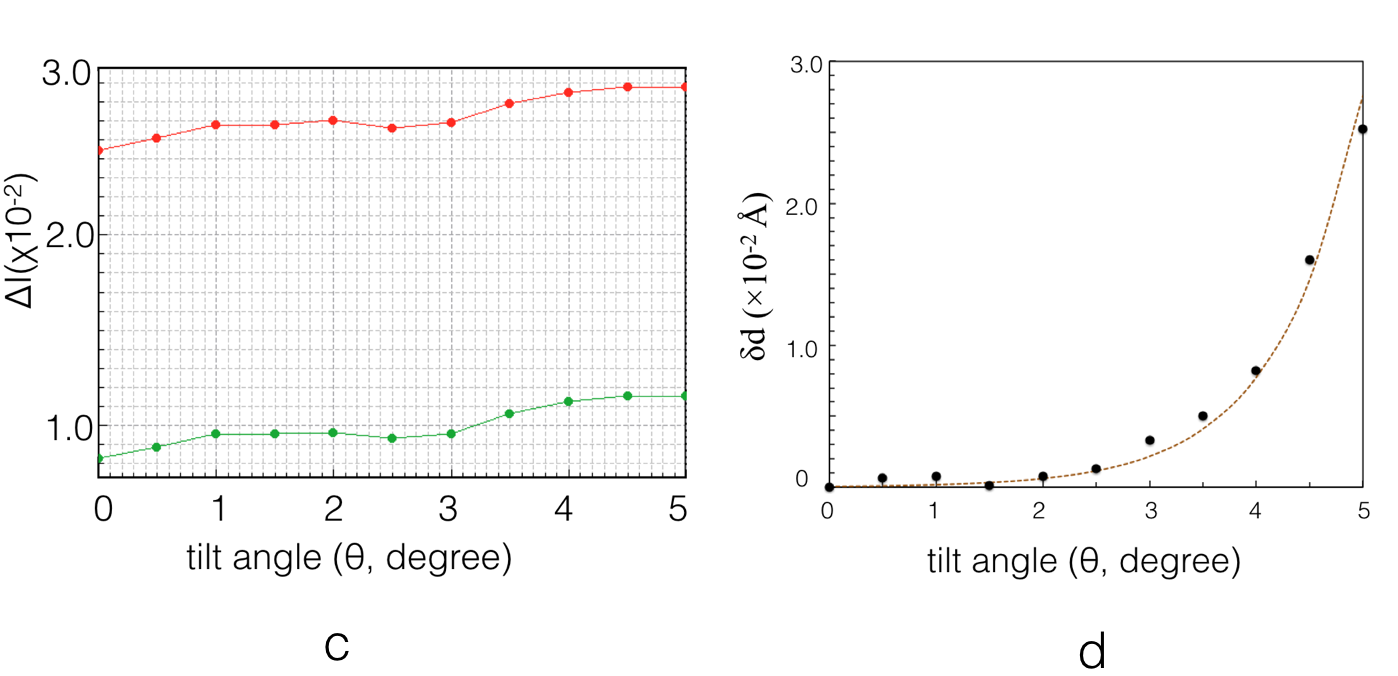


**Extended data Fig. 3.** Simulated intensity of a carbon atom in a mono/bilayer graphene sheet calculated using the multislice method as a function of tilt angle. The intensity is extracted at the central pixel of the atom. **(a)** tilt angle = 0° **(b)** tilt angle = 5°. The green circles mark the carbon atoms in the bottom layer and the red circles the atoms in the top layer. The blue circles mark overlapping atoms in the top and bottom layers. The top and bottom atom positions (green and red atoms with arrows in (b)) are displaced from the center of the hexagonal rings at a tilt of 5 °. **(c)** Simulated intensities *vs.* tilt angle for atoms in the top layer (red) and bottom layer (green). ΔΙ values are calculated in the absence of residual aberrations. **(d)** Error in layer spacing, δd=d_cal_-d_ideal_ as a function of tilt angle.


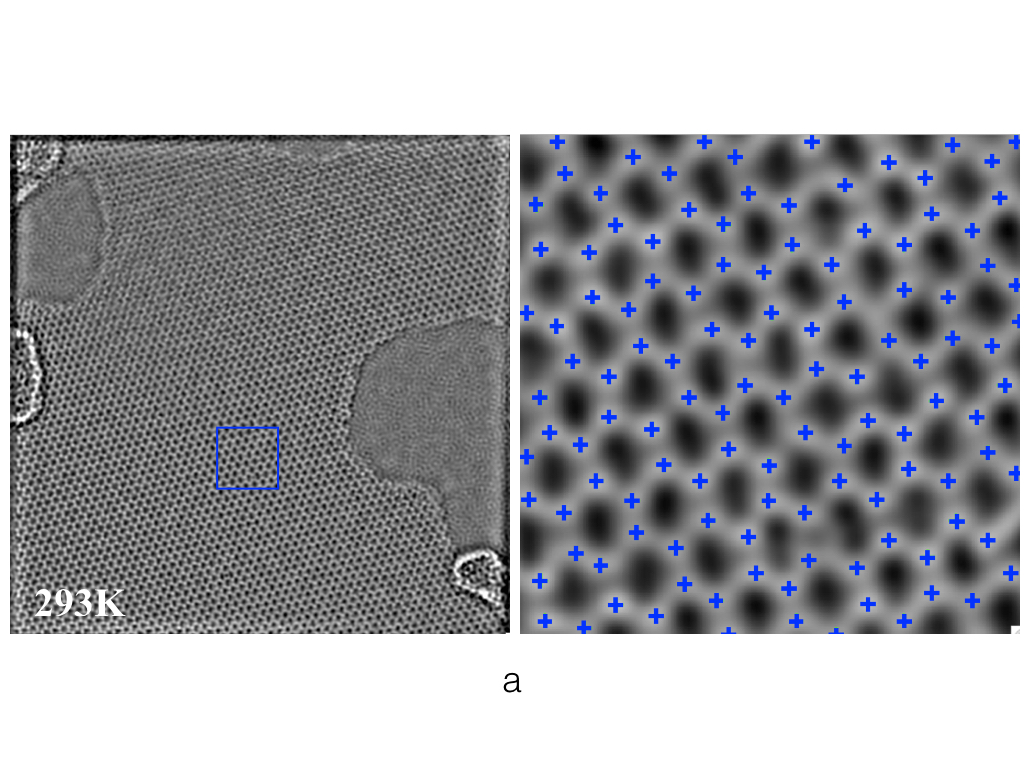


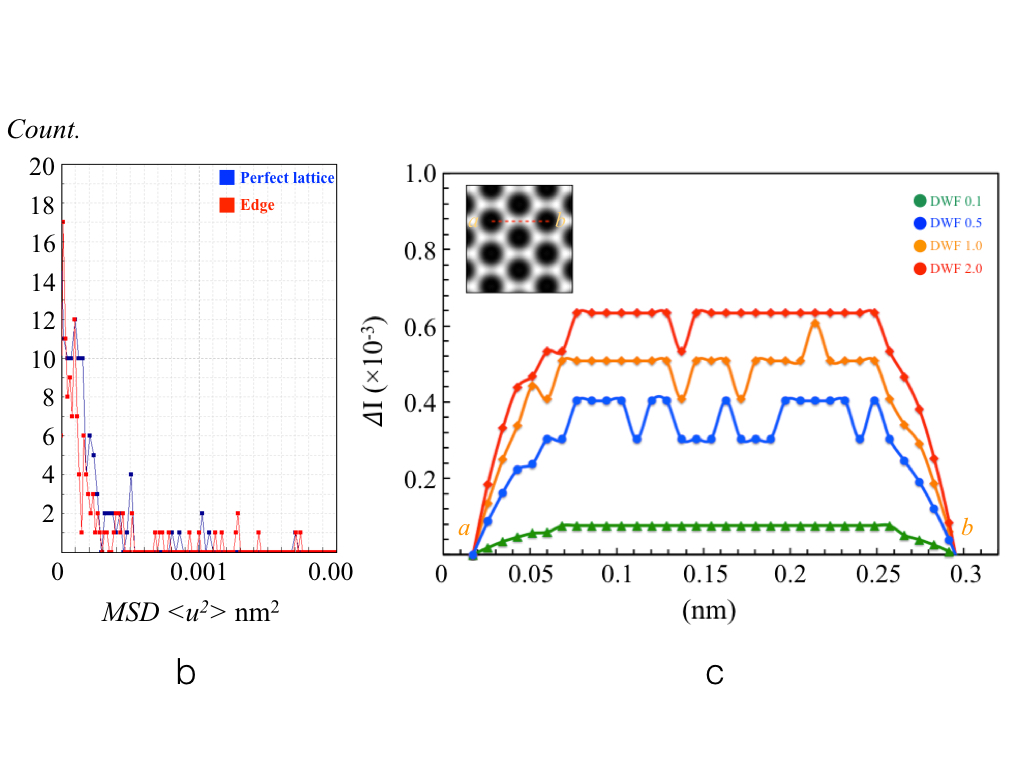


**Extended data Fig. 4. (a)** Phase of exit waves at a temperature of 293K (left) and magnified view for phase image in the boxed region (right). **(b)** Blue: Histogram of <u^2^> measured from atoms in pristine hexagonal rings (190 atoms in the square in (a)) and Red: Histogram of <u^2^> from edge atoms (130 atoms) **(c)** Difference in simulated intensity profiles δ(ΔI) across a C-C atom pair as indicated inset for different Debye-Waller factors (DWF); 0.1(green), 0.5(blue), 1.0(orange), and 2.0(red). The quantitatively effect of variations in the DWF on the image intensity are measured from the same C-C atom pair, shown between point *a* to *b* in the inset image.


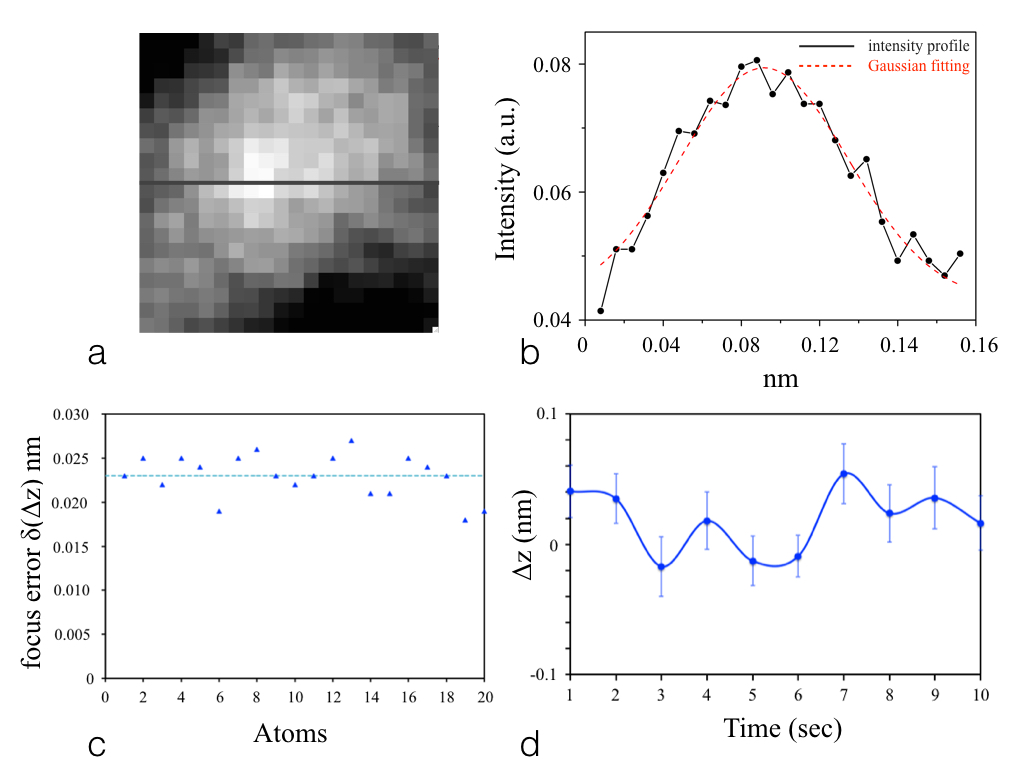


**Extended data Fig. 5. (a)** Experimental image of single carbon atom. **(b)** Intensity profile of the black line across this single carbon in (a) and the simulated Gaussian fitting (red dash line). The δ(ΔΙ), δ(ΔΙ)=ΔΙ_exp_-ΔΙ_ideal_, caused by the noise are calculated from the dismiss between experimental and ideal intensity value. The error in the z-height δ(Δz) can be calculated using equation (1). **(c)** Experimental measurements of vertical displacements for 20 atoms (blue triangles) together with their average (light blue dash line) giving a measurement error of 23pm. **(d)** The time dependent vibration in z-direction of a single atom in time-series images.


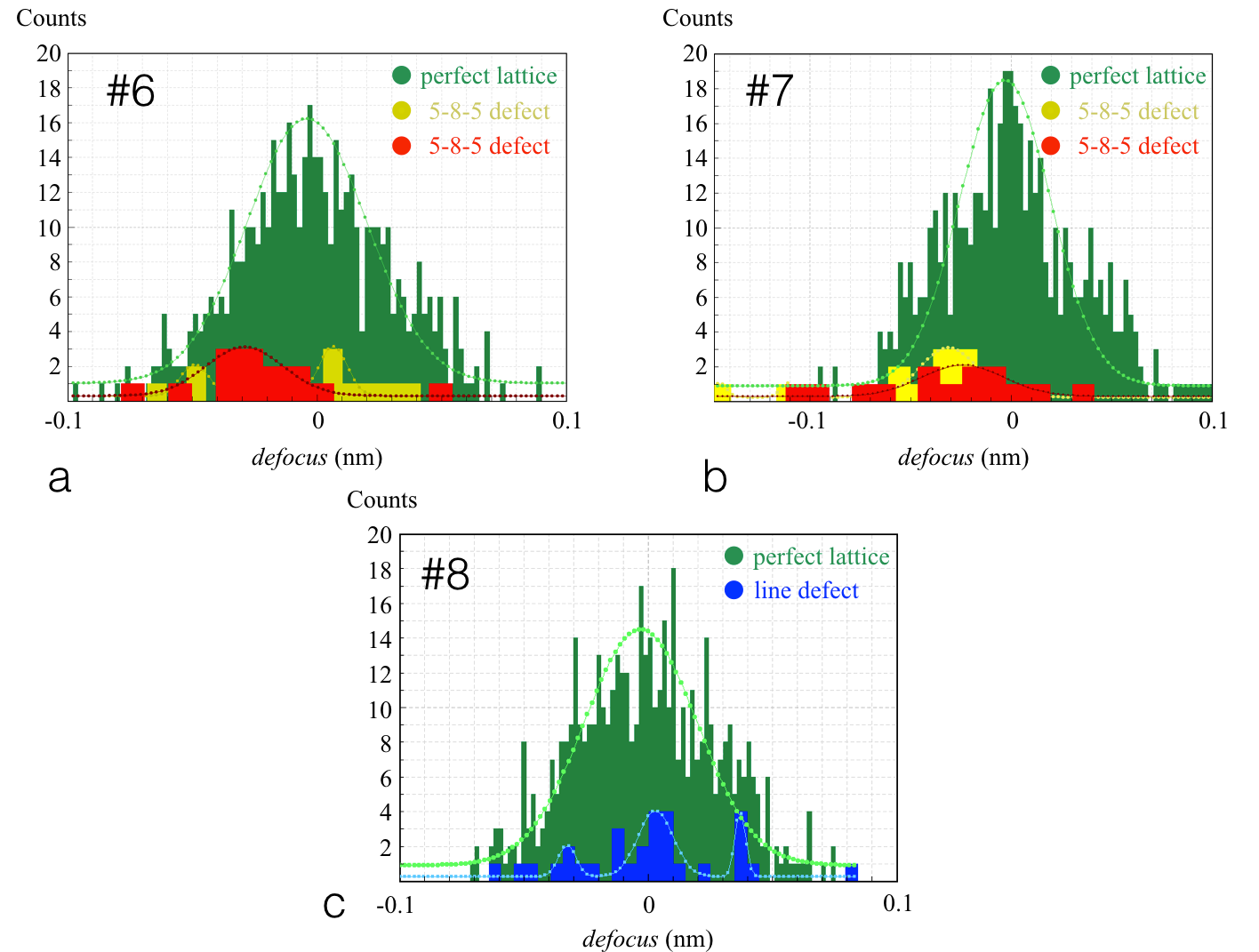


**Extended data Fig. 6.** Histogram of z-heights calculated from images **(a)** #6, **(b)** #7 and **(c)** #8 in the time series (Fig. 1). The results include z-heights of all atoms in each image. The z-heights associated with the hexagonal rings are displayed in green, while the z-heights associated with various defects are displayed in red or yellow (5-8-5 defects) and blue (5-8-4-8-4-8-5 line defects see also, extended data fig. 1).


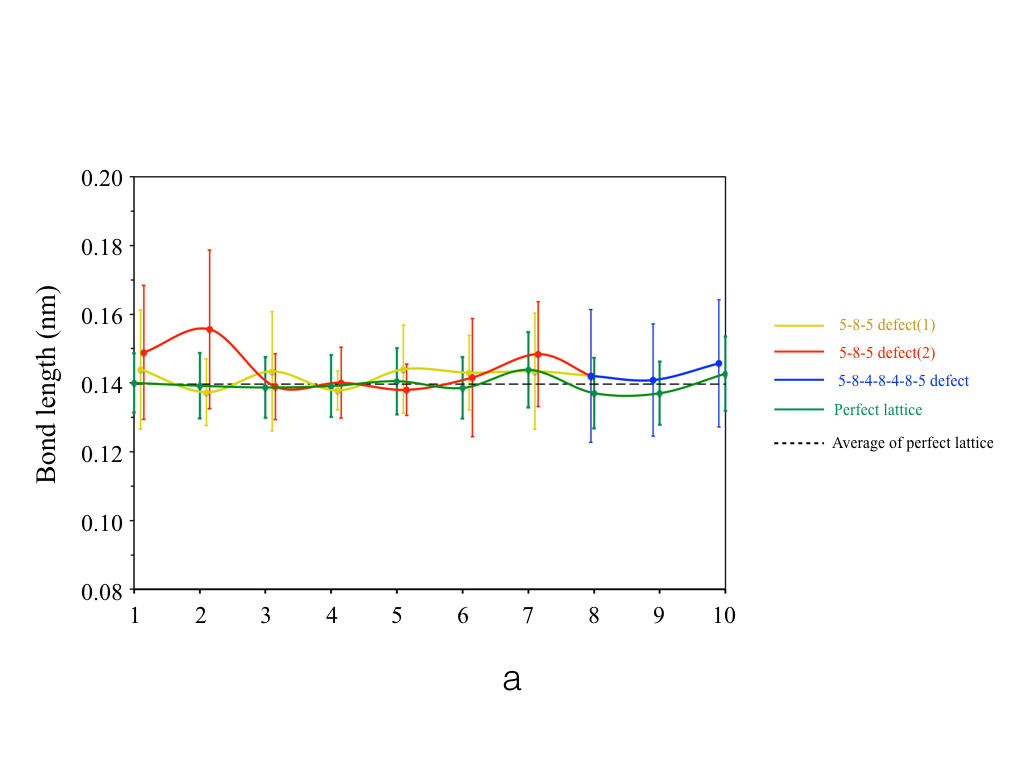


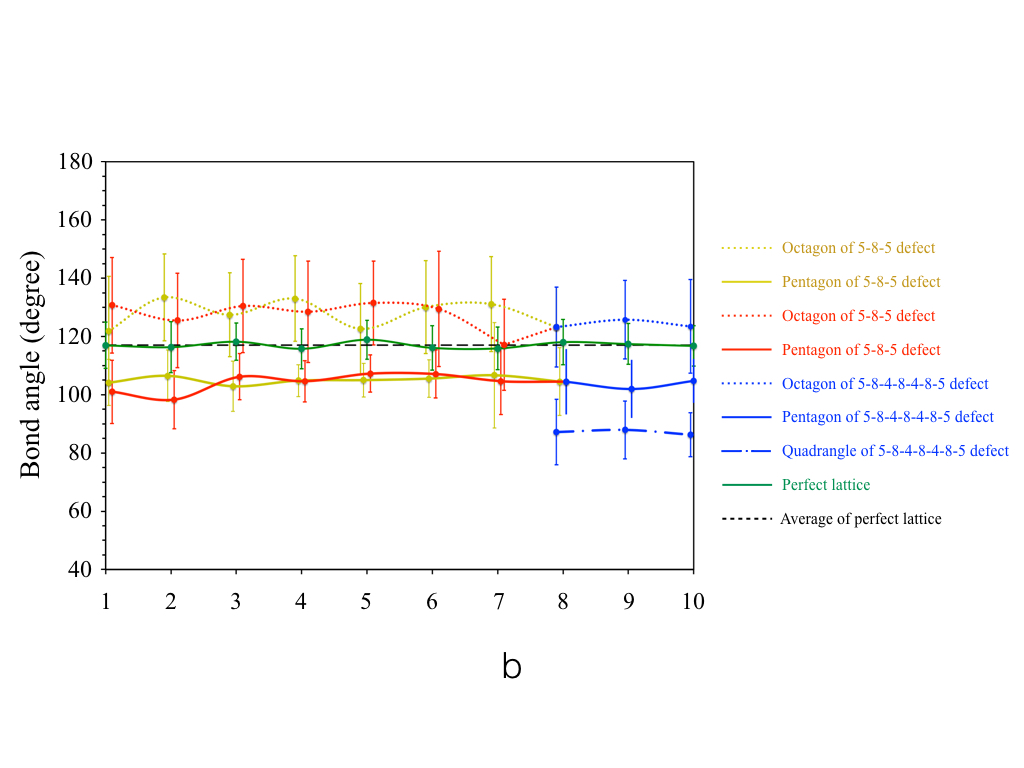


**Extended data Fig. 7.** Changes in **(a)** bond lengths and **(b)** bond angles at each C-C pair compared to those of the perfect graphene lattice for each frame in the time sequence of images (Fig. 1). (a) curves showing bond length variations. The bond length of an ideal C-C distance in graphene 0.14nm is shown as a black dashed line. The measured values of bond lengths averaged from hexagonal rings, 5-8-5 defects and 5-8-4-8-4-8-5 line defects are also shown in different colours. The measured bond angles in the pentagon, octagon and quadrangle are different, and were averaged separately. (b) bond angles in the octagon, pentagon and quadrangle shown respectively as a dashed curve, a solid curve and a broken curve with different defects indicated by different colors. The bond lengths and angles around the 8-fold rings (octagons) are distorted compared to the perfect lattice, consistent with the defect region accommodating elastic strain.


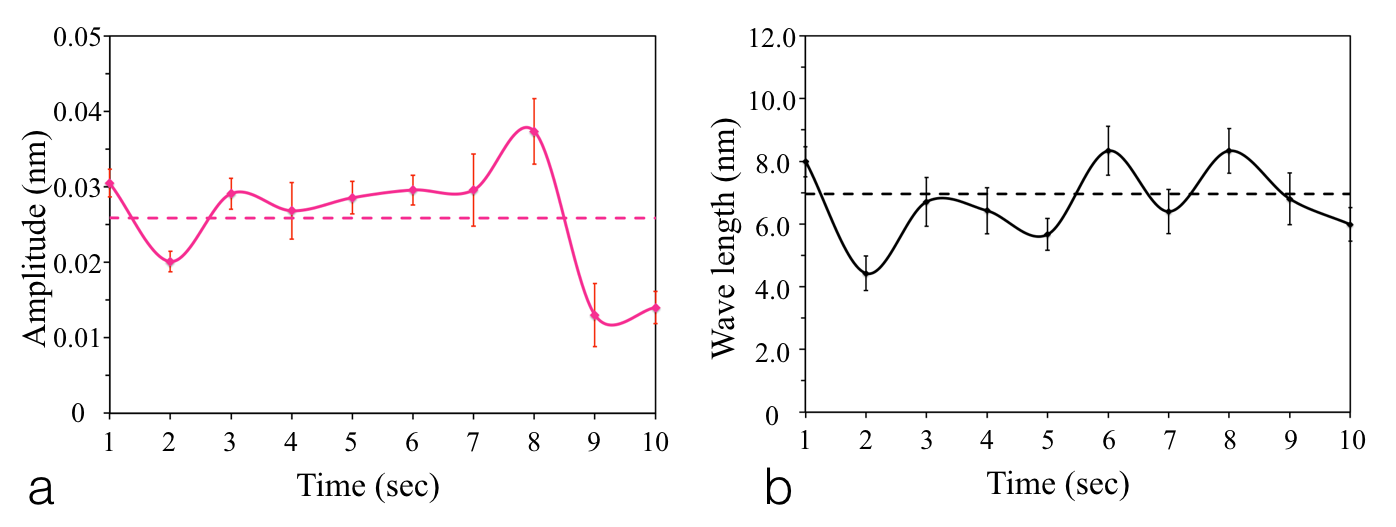


**Extended data Fig. 8.** Oscillations of a rippling graphene monolayer as a function of time for the 10 member image series described in the text. (a)amplitude=0.03±0.005nm (b) wavelength=6.96±0.66nm respectively.
